# Impact of Syndesmotic Screw Removal on Quality of Life, Mobility, and Daily Living Activities in Patients Post Distal Tibiofibular Diastasis Repair

**DOI:** 10.3390/medicina59122048

**Published:** 2023-11-21

**Authors:** Isabella-Ionela Sanda, Samer Hosin, Dinu Vermesan, Bogdan Deleanu, Daniel Pop, Dan Crisan, Musab Al-Qatawneh, Mihai Mioc, Radu Prejbeanu, Ovidiu Rosca

**Affiliations:** 1 Doctoral School, “Victor Babes” University of Medicine and Pharmacy Timisoara, 300041 Timisoara, Romania; isabella.sanda@umft.ro; 2 Department of Laboratory Medicine, “Victor Babes” University of Medicine and Pharmacy Timisoara, Eftimie Murgu Square 2, 300041 Timisoara, Romania; 3 Department of Orthopedics, “Victor Babes” University of Medicine and Pharmacy Timisoara, 300041 Timisoara, Romania; dinu@vermesan.ro (D.V.); bogdandeleanu@yahoo.com (B.D.); daniellaurentiupop@yahoo.com (D.P.); crisan.dan@gmail.com (D.C.); msb898@gmail.com (M.A.-Q.); mihaillazarmioc@gmail.com (M.M.); raduprejbeanu@gmail.com (R.P.); 4 Department of Infectious Diseases, “Victor Babes” University of Medicine and Pharmacy Timisoara, Eftimie Murgu Square 2, 300041 Timisoara, Romania; ovidiu.rosca@umft.ro

**Keywords:** ankle injuries, tibiofibular ankle syndesmosis, ankle fractures, quality of life

## Abstract

*Background and Objectives:* While numerous studies have been conducted on syndesmotic screw management following distal tibiofibular diastasis repair, a clear consensus remains unclear. This research aims to evaluate whether the postoperative removal of syndesmotic screws leads to improved patient outcomes, specifically in quality of life, mobility, and daily living activities, and whether it offers a cost-effective solution. *Materials and Methods:* Patients with a history of unimalleolar or bimalleolar ankle fractures, classified according to the Danis–Weber and Lauge–Hansen systems, were included. Comprehensive evaluations were made via standardized questionnaires like the SF-36 Health Survey, HADS, and WHOQOL-BREF, distributed approximately 2 months post surgery. A total of 93 patients underwent syndesmotic screw removal while 51 retained the screws (conservative approach). *Results:* Patients who underwent screw removal reported superior satisfaction in mobility, with a score of 7.8, compared to 6.7 in the conservative approach (*p* = 0.018). Similarly, their ability to perform daily activities scored 8.1, higher than the 6.5 from the conservative cohort (*p* < 0.001). Pain levels were also more favorable in the screw removal group, with a score of 5.3 against 6.8 in the conservative group (*p* = 0.003). On the SF-36 physical domain, the screw removal group achieved a mean score of 55.9 versus 53.3 for the conservative group (*p* = 0.027). Notably, the HADS anxiety subscale highlighted reduced anxiety levels in the screw removal cohort with a mean score of 5.8 against 7.3 in the conservative group (*p* = 0.006). However, overall quality of life and recommendations to others showed no significant difference between the groups. *Conclusions:* Syndesmotic screw removal postoperatively leads to marked improvements in patients’ mobility, daily activity abilities, and reduced postoperative pain and anxiety levels. However, overall quality of life was similar between the two approaches. The findings offer valuable insights for orthopedic decision making and patient-centered care concerning the management of syndesmotic screws after distal tibiofibular diastasis repair.

## 1. Introduction

Distal tibiofibular syndesmosis, a fibrous joint connecting the tibia and fibula just above the ankle, is integral for weight-bearing and walking [1]. The unique arrangement of ligaments in this region grants stability and permits necessary motion between the two bones [2]. Distal tibiofibular diastasis, which denotes a separation or injury to this joint, can critically compromise ankle function [3,4]. Indeed, according to existing data, such injuries represent approximately 10% of all ankle traumas, with the majority resulting from high-energy mechanisms like falls or motor vehicle accidents [5,6].

Syndesmotic screw fixation has become the gold standard for surgically managing the tibiofibular diastasis, even though other methods such as suture buttons exist and offer good results [7]. By using screws to temporarily hold the tibia and fibula together, this technique aims to ensure proper alignment and healing. Yet, the procedure is not devoid of controversy, primarily surrounding the decision to retain or remove the screws [8,9,10]. The school of thought supporting retention cites reduced reoperation rates and fewer associated complications [11], whereas proponents for removal argue that it leads to better joint function and decreases potential hardware complications [12].

Notably, the clinical implications of the decision to remove or retain the syndesmotic screw after ankle fixation weigh heavily on patient-reported outcomes [13]. Several patients are likely to express dissatisfaction following syndesmotic screw fixation, the contributing factors often including the medical intervention itself, post-removal pain, decreased range of motion, and limitations in daily activities [14]. Moreover, bone manipulation and generally orthopedic interventions increase the risk for infections that are difficult to treat, such as osteomyelitis [15,16]; thus, some patients may prefer the conservative approach. Some of the literature indicates that patients experience a tangible improvement in these domains after screw removal, although contradictory reports suggest that outcomes might be independent of the screw’s presence [17,18]. On the other side, patients that prefer to retain the syndesmotic screw might report lower anxiety levels associated with multiple medical visits and the intervention itself, but they might also have a higher comorbidity index; therefore preferring a more conservative approach [19,20].

The impact of syndesmotic screw fixation on the mechanical properties of bone, particularly its quality and quantity, is an aspect that warrants deeper exploration. Studies on prosthetic designs, such as those highlighted in the referenced works, reveal the significance of balancing material strength and flexibility under varied load conditions. In the context of hand prostheses, the emphasis on lightweight yet sturdy materials to improve functionality and aesthetics [21] mirrors the necessity in ankle surgery to find a balance between stability and the natural movement of bones. Similarly, the review of torsional loads in various engineering applications [22] underscores the importance of considering multidimensional stress factors, which could inform the approach to managing the rotational and axial stresses exerted on the ankle joint post surgery. Furthermore, insights from prosthetic acceptance studies [23], emphasizing the need for adaptability to individual needs, suggest a parallel in surgical decision making, where individual variations in bone structure and healing capacity may influence the choice between screw retention and removal.

Moreover, the economic aspects of this debate are far-reaching. Additional operations for screw removal not only incur direct medical costs but also encompass indirect expenses like patient work absence and rehabilitation. Analysis indicates that the cost of syndesmotic screw removal surgeries averaged EUR 900, compared to only EUR 400 for conservative management [24], a figure which further increases when accounting for the 10%–20% complication rate associated with such procedures [25].

Despite numerous studies on the topic, a consensus remains elusive [26]. Both the orthopedic community and patients stand to benefit from a comprehensive evaluation that not only delves into clinical outcomes but also critically assesses the cost-effectiveness and long-term implications of the decision to remove or retain syndesmotic screws. Building on this background, our study posits that postoperative syndesmotic screw removal yields significant improvements in patients’ quality of life, mobility, and ability to perform daily living activities. Furthermore, when the broader economic and societal implications are considered, screw removal may represent a more cost-effective strategy for patient management. The chief aim of this research is to conclusively evaluate these hypotheses and provide an evidence-based recommendation for the management of syndesmotic screws following distal tibiofibular diastasis repair.

## 2. Materials and Methods

### 2.1. Study Design and Settings

The present study employed a cross-sectional design; it was conducted at the University Clinic of Orthopedics affiliated with the “Victor Babes” University of Medicine and Pharmacy in Timisoara. Utilizing the clinic’s inpatient population database, we identified relevant demographic information and other pertinent clinical data from both digital and paper records. All patient data were safeguarded according to existing privacy laws and accessed by certified physicians and healthcare professionals participating in this research. The orthopedic clinic operates under the Local Commission of Ethics regulations, according to the Article 167 of Law No. 95/2006, Art. 28, Chapter VIII of Order 904/2006; the EU GCP Directives 2005/28/EC; and the International Conference on Harmonisation of Technical Requirements for Registration of Pharmaceuticals for Human Use.

### 2.2. Participant Selection and Sample Collection

Eligible participants included those with a history of unimalleolar or bimalleolar ankle fractures, identified using International Classification of Diseases (ICD-10) diagnosis codes [27]. The fractures’ classifications were, according to the Danis–Weber system, unstable type C [28]. Additionally, the Lauge–Hansen grading system further classified the fractures as SER—supination external rotation fracture; PER—pronation external rotation fracture; SA—supination adduction; and PA—pronation abduction. Other inclusion criteria encompassed patients aged 18 and above with comprehensive medical records and consent for participation. Any missing critical data or absence of a consent form resulted in exclusion from the current study. Patients who developed orthopedic complications were not considered for inclusion. All patients underwent postoperative rehabilitation.

### 2.3. Data Acquisition and Surveys

For an in-depth understanding of patients’ postoperative experiences and quality of life, several standardized questionnaires were provided. All surveys were delivered approximately 2 months after the surgical intervention for distal tibiofibular diastasis repair for the group of patients with conservative management leaving the talofibular screw intact, and 2 months after screw removal for the other group. The SF-36 Health Survey [29] was important in assessing the quality of life, spanning a spectrum of health dimensions, from physical functioning to emotional well-being. The HADS (Hospital Anxiety and Depression Scale) [30] was particularly insightful in shedding light on the mental health aspects, determining the severity of both anxiety and depressive symptoms among participants. The WHOQOL-BREF [31], comprising 26 questions, served as a broader tool to appraise the overall quality of life. In addition to these, an unstandardized survey with 8 specific questions was utilized to determine other areas not covered by the standardized questionnaires, ensuring a holistic patient perspective was captured.

Moreover, the study investigated a range of patient variables, comprising the patients’ age with and distinctions based on sex and body mass index, especially identifying those with a BMI over 25.0 kg/m^2^ and classifying them as overweight. The environmental and socioeconomic conditions of the participants were recorded by noting their areas of residence, emphasizing those from urban areas. Marital status was considered, and economic conditions were elucidated with an assessment of participants earning an average or above-average income. Educational achievements were recorded by highlighting those with higher education, and employment status focused on those unemployed. Lifestyle habits, which can profoundly impact recovery and rehabilitation, were not overlooked, documenting frequent alcohol consumers and regular smokers. A significant clinical variable was the Charlson Comorbidity Index (CCI), with special attention given to those with a score greater than 2. Regarding the clinical specifics, fracture types were categorized into unimalleolar and bimalleolar. Further classification was conducted based on the Lauge–Hansen system, differentiating fractures into SER (supination external rotation), PER (pronation external rotation), SA (supination adduction), and PA (pronation abduction).

### 2.4. Statistical Analysis

Data management and analysis were conducted utilizing the statistical software SPSS version 26.0 (SPSS Inc., Chicago, IL, USA). The sample size was calculated based on a convenience sampling method, with a minimum requirement for statistical power of 129 respondents at a 95% confidence level and 5% margin of error. Continuous variables were represented as mean ± standard deviation (SD), while categorical variables were expressed in terms of frequencies and percentages. To analyze the changes between more than two means of continuous variables, Student’s *t*-test was utilized. The Chi-squared test was utilized for the categorical variables. A multivariate regression analysis was performed to determine the risk factors for influenced quality of life. A *p*-value threshold of less than 0.05 was set for statistical significance. All results were double-checked to ensure accuracy and reliability.

## 3. Results

From the total sample, 93 patients underwent syndesmotic screw removal while 51 were managed with a conservative approach. Age distribution was similar between the two groups, with the screw removal cohort averaging 32.8 ± years and the conservative approach cohort averaging 33.5 years; this difference was not statistically significant (*p* = 0.759). In terms of gender distribution, 60.2% of the screw removal group were men, compared to 45.1% in the conservative group; this observed difference approached significance but did not reach it (*p* = 0.081). The proportion of overweight individuals, defined by a BMI of greater than 25.0 kg/m^2^, was slightly higher in the conservative approach group, at 47.1%, than in the screw removal group, at 38.7%, though the difference was not statistically significant (*p* = 0.331).

Most of the background characteristics such as area of residence (urban vs. rural), relationship status (married vs. other), income levels, education levels, and employment status revealed no significant differences between the two groups. The same was observed for habits such as frequent alcohol consumption and smoking. Moreover, there was no significant difference in the Charlson Comorbidity Index (CCI) > 2 between the two groups. Fracture type, categorized as unimalleolar or bimalleolar, showed similar distributions between the groups and did not present statistically significant differences (*p* = 0.762). The Lauge–Hansen classification, which differentiates fractures based on patterns such as supination external rotation (SER), pronation external rotation (PER), supination adduction (SA), and pronation abduction (PA), also did not show significant differences between the groups (*p* = 0.185), as presented in Table 1.

Participants were asked about their satisfaction with overall mobility since the surgical procedure. Those who underwent syndesmotic screw removal reported an average satisfaction score of 7.8, which was significantly higher than the 6.7 reported by the conservative approach cohort (*p* = 0.018). When questioned on the perceived impact of the screw removal (or retention) on their ability to perform daily living activities, the screw removal group rated this aspect at 8.1, significantly higher than the 6.5 from the conservative approach group (*p* < 0.001). In terms of pain or discomfort experienced post surgery, the screw removal group averaged a score of 5.3, which was significantly lower, indicating less pain or discomfort, than the 6.8 reported by the conservative approach group (*p* = 0.003).

However, not all results favored the screw removal procedure. When asked if they would recommend their respective treatment approach to someone else with a similar condition, both groups displayed almost equal confidence, with the screw removal group scoring 7.6 and the conservative approach scoring 7.4 (*p* = 0.591). Similarly, there was no significant difference between the two groups when asked about the impact of the surgical procedure on their quality of life: the screw removal group scored 6.9, and the conservative approach scored 7.1 (*p* = 0.714).

Nevertheless, participants’ confidence in their decision varied significantly. Those who opted for screw removal expressed higher confidence in their choice, scoring 8.9, compared to 7.8 by those who chose conservative treatment (*p* = 0.013). The patients’ perception of limitations in daily activities due to the surgical procedure also showed significant differences, with the screw removal group averaging a score of 5.6 and the conservative approach group scoring higher at 6.9 (*p* = 0.016), indicating more perceived limitations. Lastly, in terms of patient information regarding the advantages and disadvantages of screw removal versus retention, the scores were close between groups, with 8.4 for the screw removal cohort and 8.0 for the conservative approach, and this difference was not statistically significant (*p* = 0.222), as presented in Table 2.

**Figure 1:**
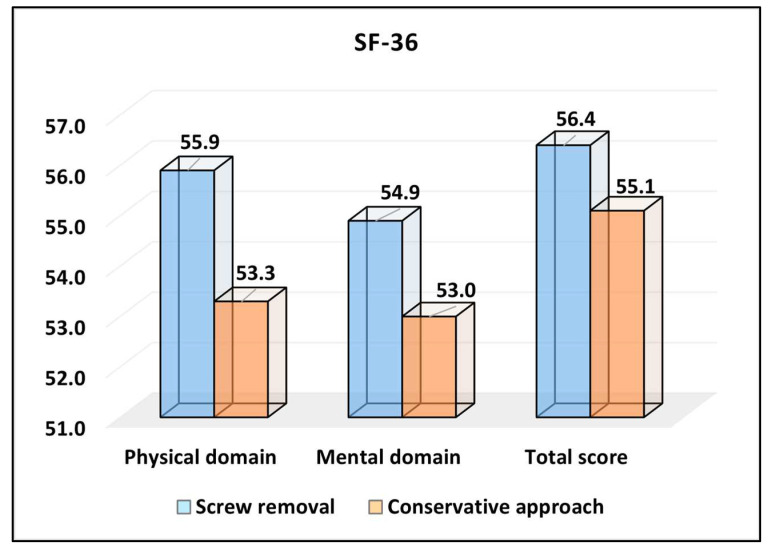
Analysis of the SF-36 questionnaire results.

For the physical domain of the SF-36 survey, patients in the screw removal group reported a mean score of 55.9, which was significantly higher than the 53.3 reported by the conservative approach group (*p* = 0.027), as seen in Table 3 and Figure 1. This suggests that those who underwent screw removal experienced a better physical health status compared to those who opted for the conservative approach. Regarding the mental domain of the survey, the difference between the groups was not statistically significant. The screw removal group scored an average of 54.9, slightly higher than the 53.0 of the conservative approach group, but the *p*-value of 0.140 indicated that this difference was not significant. Therefore, both groups had comparable mental health statuses postoperatively. The total score on the SF-36, which encompasses both physical and mental health domains, showed a mean of 56.4 for the screw removal group and 55.1 for the conservative approach group. This difference was not statistically significant (*p* = 0.349), implying that, when considering overall health status and quality of life, the two groups were largely comparable.

The physical domain of the WHOQOL-BREF survey showed that the screw removal group had a mean score of 64.8, which was higher than the 60.9 ± 11.6 reported by the conservative approach group. Although this difference suggests a better physical quality of life for the screw removal group, the *p*-value of 0.064 indicated that this difference was marginally outside the conventional threshold of statistical significance. For the mental domain, the conservative approach group reported a mean score of 66.4, which was higher than the 62.3 from the screw removal group. This difference, with a *p*-value of 0.082, was also marginally nonsignificant, suggesting that there might be a trend towards a better mental quality of life in the conservative approach group, but this was not conclusively demonstrated.

**Figure 2:**
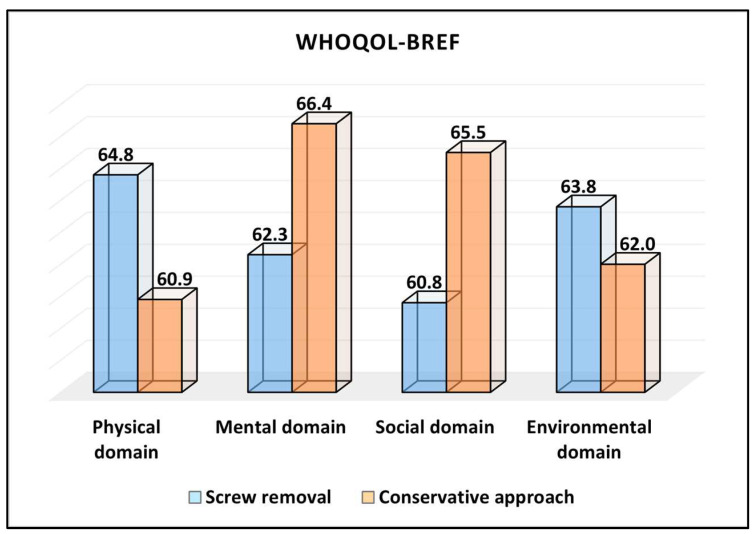
Average values of domain scores on the WHOQOL-BREF questionnaire.

In the social domain, there was a statistically significant difference between the two groups. The conservative approach group had a higher average score of 65.5 than the 60.8 from the screw removal group, with a *p*-value of 0.039, as presented in Table 4 and Figure 2. This indicates that patients in the conservative approach group reported a better social quality of life postoperatively. The environmental domain showed no significant difference between the two groups. The screw removal group reported a score of 63.8, while the conservative approach group reported 62.0, with a *p*-value of 0.399, indicating that the environmental quality of life was comparable between the two treatments.

**Figure 3:**
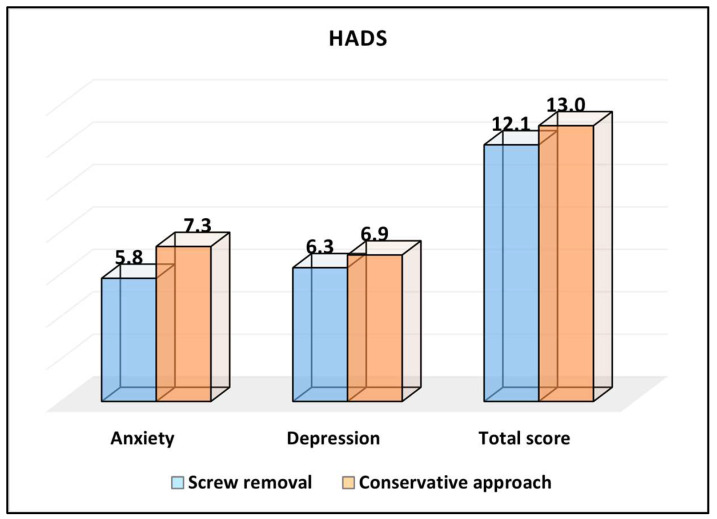
Analysis of the HADS questionnaire results.

Starting with the anxiety subscale of the HADS, the group that underwent screw removal reported a mean score of 5.8. In contrast, the conservative approach group had a higher mean score of 7.3. This difference was statistically significant, with a *p*-value of 0.006, suggesting that patients who underwent screw removal experienced, on average, lower levels of anxiety compared to those who followed the conservative approach. Regarding the depression subscale, the screw removal group had a mean score of 6.3, which was marginally lower than the 6.9 reported by the conservative approach group. However, with a *p*-value of 0.109, this difference was not statistically significant, indicating that both groups had similar levels of depression postoperatively. When considering the total HADS score, which combines both anxiety and depression components, the screw removal group showed a mean score of 12.1. This was slightly lower than the 13.0 from the conservative approach group. Nevertheless, the difference was not statistically significant, as evidenced by a *p*-value of 0.252, as described in Table 5 and Figure 3.

Female patients, in contrast to males, demonstrated a 0.8 hazard ratio (HR), which means they had a 20% reduced risk of presenting with a low score on the SF-36 physical domain. This relationship bore statistical significance, given that the 95% confidence interval (CI) spanned from 0.6 to 1.1 and was solidified with a *p*-value of 0.014. Consequently, being female could conceivably be associated with a better perception of physical health in this specific patient group. Furthermore, anxiety levels, as per the HADS scale, revealed an intriguing pattern. For every unit increase in anxiety score, the likelihood of a lower score in the SF-36 physical domain surged by 60% (HR = 1.6), a finding corroborated by a 95% CI ranging from 1.2 to 3.1 and a significant *p*-value of 0.006. This suggests that elevated anxiety levels might substantially compromise the perceived physical quality of life.

**Figure 4:**
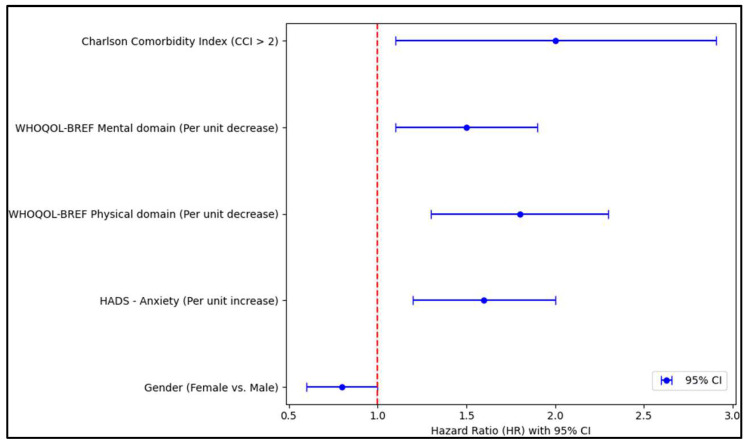
Regression analysis results.

The WHOQOL-BREF, employed to gauge quality of life across various domains, also unveiled noteworthy observations. Specifically, each unit elevation in the physical domain score corresponded to an 80% amplified risk (HR = 1.8) of a depressed SF-36 physical score. This relationship was robust, denoted by its CI from 1.3 to 2.5 and a highly significant *p*-value of less than 0.001. The mental domain of the same scale indicated that every unit increase was linked to a 50% heightened risk (HR = 1.5) of a lower SF-36 physical score, with its 95% CI spanning 1.1 to 2.8 and a *p*-value of 0.013, as seen in Table 6 and Figure 4. Moreover, patients with a Charlson Comorbidity Index (CCI) exceeding 2 were found to be twice as likely (HR = 2.0) to report lower scores on the SF-36 physical domain. This association’s strength is highlighted by the CI, which ranged from 1.1 to 3.6, and a *p*-value of 0.024, indicating the potential influence of multiple comorbidities on the perceived physical health quality of life.

## 4. Discussion

### 4.1. Literature Findings

The debate surrounding the optimal management of syndesmotic screws post distal tibiofibular diastasis repair continues to perplex the orthopedic community [32,33]. Grounded in this context, our study sought to elucidate the impact of screw removal on quality of life, mobility, and activities of daily living in postoperative patients. The findings suggest that there are indeed significant differences in specific patient-reported outcomes depending on whether the syndesmotic screws are removed or retained.

When investigating mobility and daily living activities post surgery, participants who underwent screw removal indicated a perceptibly improved satisfaction score in contrast to those who favored the conservative approach. This result aligns with the theory that screw removal may alleviate some of the mechanical restrictions that syndesmotic screws could impose [13,34]. Such outcomes underscore the potential advantages of screw removal in fostering enhanced mobility, a cornerstone for improved rehabilitation and overall quality of life. However, even though the reduced pain or discomfort reported by the screw removal group further solidifies this stance, screw removal can also determine various complications such as infection of early loss of reduction, therefore worsening the quality of life and mobility [13].

However, our study also highlighted areas where there was little discernment between the two groups. For instance, both groups were equivocal in their willingness to recommend their respective treatments to peers, suggesting that despite the measurable differences in certain outcomes, overall satisfaction may be influenced by various factors not exclusively related to the presence or absence of the screw. Intriguingly, the assessment of overall quality of life did not demonstrate a statistically significant difference between the groups. This parity, when juxtaposed against the marked differences in mobility and pain scores, might reflect the multi-faceted nature of the quality of life construct. It emphasizes that while physical factors play a pivotal role, psychological, social, and environmental variables also hold considerable weight.

Diving deeper into the psychological aspects, the anxiety subscale of the HADS presented an illuminating pattern. The group that underwent screw removal reported significantly reduced anxiety levels compared to their counterparts. Such findings could be indicative of the mental relief afforded by the removal of the syndesmotic screw, perhaps due to a perceived return to a more ‘natural’ state or the elimination of potential future surgery for screw-related complications. In contrast, levels of depression did not differ significantly between the groups, highlighting that while interventions might ameliorate certain psychological aspects, they may not offer relief for all mental health concerns postoperatively.

Our findings also resonate with a broader context when scrutinizing the gender-based disparities. Female participants seemed to have a better perception of physical health, as indicated by the SF-36 physical domain, suggesting possible gender-specific differences in postoperative adaptation or pain perception. Nevertheless, this is in contrast with the existing literature showing that women have lower thresholds to pain than their male counterparts [35,36]. This result reiterates the need for personalized care tailored to specific demographic subsets. Moreover, the profound influence of anxiety on perceived physical quality of life underscores the intrinsic link between mental and physical health in postoperative rehabilitation.

Drawing parallels with other studies, it is noteworthy that the tangible benefits of screw removal affect mobility and pain, thus aligning with the previous literature and increasing the reliability of our findings. Nevertheless, it should not be omitted that other studies report no significant mobility changes after syndesmotic screw removal [37]. While the debate persists, this research, by critically evaluating both clinical outcomes and broader economic implications, casts a more holistic light on the decision making process surrounding syndesmotic screw management.

It is imperative to consider the potential effects of syndesmotic screw removal on bone architecture, which bears significant relevance for orthopedic decision making and patient-centered care. The mechanical integrity of the distal tibiofibular joint, influenced by factors such as bone density, shape, and overall architecture, is crucial for determining the success of syndesmotic screw procedures. The current literature, although not entirely conclusive, suggests that screw removal can influence the microstructure and mechanical properties of the bone, potentially impacting its ability to withstand normal physiological loads and stresses [38]. This aspect is particularly important in the context of long-term bone health and the prevention of osteoarthritis or other degenerative conditions. The decision to remove or retain the syndesmotic screw, therefore, must be made with a comprehensive understanding of these biomechanical implications, alongside patient-specific factors such as age, bone quality, activity level, and personal preferences. Our study’s findings, emphasizing the importance of physical and psychological outcomes, should thus be contextualized within this broader framework of bone architecture considerations to guide more nuanced and individualized patient care strategies.

### 4.2. Study Limitations

The current study possesses several inherent limitations. Firstly, the cross-sectional design used restricts the capability to infer causal relationships between variables, limiting our conclusions to associations observed at one specific point in time. While the study relied on the clinic’s inpatient population database for demographic and clinical data, the possibility of inconsistencies between digital and paper records cannot be completely ruled out. The sample size, determined through a convenience sampling method, might not wholly represent the broader population of individuals with unimalleolar or bimalleolar ankle fractures, potentially introducing selection bias. The exclusion of patients with missing data or those who developed orthopedic complications could lead to the omission of certain clinical scenarios from our analysis. Another notable limitation of our study is the relatively short follow-up period of approximately two months post surgery, which may not sufficiently capture long-term outcomes and potential complications associated with distal tibiofibular diastasis repair, thereby limiting our ability to comprehensively assess the enduring effects of the treatment strategies employed. Moreover, even though a variety of standardized questionnaires were utilized, the reliance on self-reported data poses a risk of recall bias. Lastly, the study’s findings are based on data from a single center, which could limit the generalizability of results to other settings or populations.

## 5. Conclusions

This study provided a comprehensive analysis of the outcomes related to the management of syndesmotic screws post distal tibiofibular diastasis repair. The postoperative removal of syndesmotic screws was found to have a favorable impact on several patient-centered outcomes. Specifically, patients who underwent screw removal exhibited enhanced mobility, superior ability to execute daily activities, and experienced reduced levels of postoperative pain and anxiety than those who adopted a conservative approach by retaining the screws. Notably, these benefits did not translate into a significant difference in the overall quality of life between the two groups. Despite these advancements in our understanding, the decision to remove or retain the screw should be personalized and tailored to individual patient needs, considering the multifaceted nature of postoperative recovery. The insights gleaned from this study augment the current orthopedic knowledge and serve as a significant reference for delivering patient-centric care in the context of distal tibiofibular diastasis repair.

## Figures and Tables

**Table 1:** Comparison of the study cohort background characteristics.

Variables	Screw Removal (*n* = 93)	Conservative Approach (*n* = 51)	*p*-Value *
Age, years	32.8 ± 12.5	33.5 ± 14.1	0.759
Sex (men, %)	56 (60.2%)	23 (45.1%)	0.081
Overweight (>25.0 kg/m^2^)	36 (38.7%)	24 (47.1%)	0.331
Area of residence (urban)	58 (62.4%)	28 (54.9%)	0.382
Relationship status (married)	62 (66.7%)	37 (72.5%)	0.466
Level of income (average or higher)	53 (57.0%)	29 (56.9%)	0.988
Level of education (higher education)	66 (71.0%)	31 (60.8%)	0.212
Occupation (employed)	51 (54.8%)	32 (62.7%)	0.358
Frequent alcohol consumption	10 (10.8%)	8 (15.7%)	0.392
Frequent smoker	25 (26.9%)	17 (33.3%)	0.415
CCI > 2	8 (8.6%)	3 (5.9%)	0.556
Fracture type			0.762
Unimalleolar	24 (25.8%)	12 (23.5%)	
Bimalleolar	49 (52.7%)	24 (47.1%)	
Lauge–Hansen classification			0.185
SER	42 (45.2%)	17 (33.3%)	
PER	30 (32.3%)	14 (27.5%)	
SA	10 (10.8%)	11 (21.6%)	
PA	11 (11.8%)	8 (15.7%)	

* Chi-squared or Fisher’s exact test; CCI—Charlson Comorbidity Index; SER—supination external rotation fracture; PER—pronation external rotation fracture; SA—supination adduction; PA—pronation abduction.

**Table 2:** Unstandardized survey results.

Questions	Screw Removal (*n* = 93)	Conservative Approach (*n* = 51)	*p*-Value *
Q1: How satisfied are you with your overall mobility since the surgical procedure?	7.8 ± 2.5	6.7 ± 2.9	0.018
Q2: To what extent do you believe the removal (or retention) of the syndesmotic screw impacted better your ability to perform daily living activities?	8.1 ± 2.1	6.5 ± 3.0	<0.001
Q3: How often do you experience pain or discomfort in the area of the surgical procedure?	5.3 ± 2.7	6.8 ± 3.2	0.003
Q4: Would you recommend the same treatment approach to someone else with a similar condition? (1 being not likely, 10 being highly likely)	7.6 ± 1.9	7.4 ± 2.6	0.591
Q5: How has this surgical procedure impacted your quality of life? (higher is better)	6.9 ± 3.3	7.1 ± 2.8	0.714
Q6: How confident are you in your decision (either removal or retention of the screw)?	8.9 ± 1.6	7.8 ± 2.4	0.013
Q7: Have you noticed any limitations in daily activities due to the surgical procedure?	5.6 ± 2.9	6.9 ± 3.3	0.016
Q8: On a scale of 1 to 10, how would you rate the information provided to you regarding the pros and cons of screw removal versus retention?	8.4 ± 1.8	8.0 ± 2.0	0.222

* Student’s *t*-test.

**Table 3:** SF-36 survey results.

Scores (Mean ± SD)	Screw Removal (*n* = 93)	Conservative Approach (*n* = 51)	*p*-Value *
SF-36—Physical	55.9 ± 6.6	53.3 ± 6.8	0.027
SF-36—Mental	54.9 ± 7.2	53.0 ± 7.6	0.140
SF-36—Total	56.4 ± 7.8	55.1 ± 8.2	0.349

* Student’s *t*-test; SD—standard deviation; SF-36—short-form survey (higher scores indicate better health status and quality of life).

**Table 4:** WHOQOL-BREF survey results.

WHOQOL-BREF (Mean ± SD)	Screw Removal (*n* = 93)	Conservative Approach (*n* = 51)	*p*-Value *
Physical domain	64.8 ± 12.2	60.9 ± 11.6	0.064
Mental domain	62.3 ± 13.8	66.4 ± 12.8	0.082
Social domain	60.8 ± 12.9	65.5 ± 13.1	0.039
Environmental domain	63.8 ± 11.6	62.0 ± 13.3	0.399

* Student’s *t*-test; SD—standard deviation; WHOQOL-BREF—brief version of the World Health Organization Quality of Life survey (higher scores indicate better quality of life).

**Table 5:** HADS survey results.

HADS (Mean ± SD)	Screw Removal (*n* = 93)	Conservative Approach (*n* = 51)	*p*-Value *
Anxiety	5.8 ± 3.4	7.3 ± 2.5	0.006
Depression	6.3 ± 2.3	6.9 ± 1.8	0.109
Total score	12.1 ± 4.6	13.0 ± 4.3	0.252

* Student’s *t*-test; SD—standard deviation; SF-36—short-form survey (higher scores indicate higher levels of anxiety or depression).

**Table 6:** Regression analysis for low SF-36 scores on the physical domain.

Independent Variables	HR—Exp (B)	95% CI	*p*-Value
Gender (Female vs. Male)	0.8	0.6–1.1	0.014
Age (Per year increase)	1.02	0.97–1.19	0.132
Overweight (>25.0 kg/m^2^)	1.4	1.0–1.9	0.072
Area of residence (Urban vs. Rural)	1.1	0.8–1.4	0.621
Relationship status (Relationship vs. Single)	0.9	0.6–1.3	0.466
Level of income (Average or higher vs. Below average)	1.2	0.9–2.6	0.201
Level of education (Higher vs. Below higher)	1.1	0.8–1.5	0.499
Occupation (Employed vs. Unemployed)	0.9	0.7–1.1	0.173
Frequent alcohol consumption (Yes vs. No)	0.8	0.6–1.2	0.091
Frequent smoker (Yes vs. No)	1.2	0.9–1.6	0.212
Fracture type (Unimalleolar vs. Bimalleolar)	1.2	0.9–2.2	0.193
HADS—Anxiety (Per unit increase)	1.6	1.2–3.1	0.006
HADS—Depression (Per unit increase)	1.1	0.9–2.0	0.218
WHOQOL-BREF Physical domain	1.8	1.3–2.5	<0.001
WHOQOL-BREF Mental domain	1.5	1.1–2.8	0.013
WHOQOL-BREF Social domain	1.6	0.9–2.2	0.095
WHOQOL-BREF Environmental domain	1.4	1.0–1.9	0.068
Charlson Comorbidity Index (CCI > 2)	2.0	1.1–3.6	0.024

HR—hazard ratio; CI—confidence interval; SF—short form; data in this table analyze both study groups.

## Data Availability

Data available on request.

## References

[1] Yuen C.P., Lui T.H. (2017). Distal Tibiofibular Syndesmosis: Anatomy, Biomechanics, Injury and Management. Open Orthop. J..

[2] Carto C., Lezak B., Varacallo M. (2023). Anatomy, Bony Pelvis and Lower Limb: Distal Tibiofibular Joint (Tibiofibular Syndesmosis). StatPearls [Internet].

[3] Pogliacomi F., De Filippo M., Casalini D., Longhi A., Tacci F., Perotta R., Pagnini F., Tocco S., Ceccarelli F. (2021). Acute syndesmotic injuries in ankle fractures: From diagnosis to treatment and current concepts. World J. Orthop..

[4] Hunt K.J. (2013). Syndesmosis injuries. Curr. Rev. Musculoskelet. Med..

[5] Magan A., Golano P., Maffulli N., Khanduja V. (2014). Evaluation and management of injuries of the tibiofibular syndesmosis. Br. Med. Bull..

[6] Cao M.-M., Zhang Y.-W., Hu S.-Y., Rui Y.-F. (2022). A systematic review of ankle fracture-dislocations: Recent update and future prospects. Front. Surg..

[7] Yawar B., Hanratty B., Asim A., Niazi A.K., Khan A.M. (2021). Suture-Button Versus Syndesmotic Screw Fixation of Ankle Fractures: A Comparative Retrospective Review Over One Year. Cureus.

[8] Corte-Real N., Caetano J. (2021). Ankle and syndesmosis instability: Consensus and controversies. EFORT Open Rev..

[9] Baxter S., Farris E., Johnson A.H., Brennan J.C., Friedmann E.M., Turcotte J.J., Keblish D.J. (2023). Transosseous Fixation of the Distal Tibiofibular Syndesmosis: Comparison of Interosseous Suture and Endobutton Across Age Groups. Cureus.

[10] Sipahioglu S., Zehir S., Isikan U.E. (2018). Syndesmotic screw fixation in tibiofibular diastasis. Niger. J. Clin. Pr..

[11] Kapadia B.H., Sabarese M.J., Chatterjee D., Aylyarov A., Zuchelli D.M., Hariri O.K., Uribe J.A., Tsai J. (2020). Evaluating success rate and comparing complications of operative techniques used to treat chronic syndesmosis injuries. J. Orthop..

[12] Walley K.C., Hofmann K.J., Velasco B.T., Kwon J.Y. (2017). Removal of Hardware After Syndesmotic Screw Fixation: A Systematic Literature Review. Foot Ankle Spéc..

[13] Schepers T. (2011). To retain or remove the syndesmotic screw: A review of literature. Arch. Orthop. Trauma Surg..

[14] Ijezie N., Fraig H., Abolaji S. (2022). Outcomes of the Routine Removal of the Syndesmotic Screw. Cureus.

[15] Zeng M., Xu Z., Song Z.Q., Li J.X., Tang Z.W., Xiao S., Wen J. (2023). Diagnosis and treatment of chronic osteomyelitis based on nanomaterials. World J. Orthop..

[16] Ribeiro M., Monteiro F.J., Ferraz M.P. (2012). Infection of orthopedic implants with emphasis on bacterial adhesion process and techniques used in studying bacterial-material interactions. Biomatter.

[17] Moon Y.J., Kim D.H., Lee K.B. (2020). Is it necessary to remove syndesmotic screw before weight-bearing ambulation?. Medicine.

[18] Schnetzke M., Vetter S.Y., Beisemann N., Swartman B., Grützner P.A., Franke J. (2016). Management of syndesmotic injuries: What is the evidence?. World J. Orthop..

[19] Kujanpää T., Jokelainen J., Auvinen J., Timonen M. (2016). Generalised anxiety disorder symptoms and utilisation of health care services. A cross-sectional study from the “Northern Finland 1966 Birth Cohort”. Scand. J. Prim. Health Care.

[20] Mao W., Shalaby R., Agyapong V.I.O. (2023). Interventions to Reduce Repeat Presentations to Hospital Emergency Departments for Mental Health Concerns: A Scoping Review of the Literature. Healthcare.

[21] Buccino F., Bunt A., Lazell A., Vergani L.M. (2022). Mechanical Design Optimization of Prosthetic Hand’s Fingers: Novel Solutions towards Weight Reduction. Materials.

[22] Buccino F., Martinoia G., Vergani L.M. (2021). Torsion—Resistant Structures: A Nature Addressed Solution. Materials.

[23] Millstein S.G., Heger H., Hunter G. (1986). Prosthetic Use in Adult Upper Limb Amputees: A Comparison of the Body Powered and Electrically Powered Prostheses. Prosthet. Orthot. Int..

[24] Hosin S., Vermesan D., Prejbeanu R., Crisan D., Al-Qatawneh M., Pop D., Mioc M., Bratosin F., Feciche B., Hemaswini K. (2022). Avoiding the Removal of Syndesmotic Screws after Distal Tibiofibular Diastasis Repair: A Benefit or a Drawback?. J. Clin. Med..

[25] Bragg J.T., Masood R.M., Spence S.S., Citron J.E., Moon A.S., Salzler M.J., Ryan S.P. (2023). Predictors of Hardware Removal in Orthopaedic Trauma Patients Undergoing Syndesmotic Ankle Fixation with Screws. Foot Ankle Orthop..

[26] Desouky O., Elseby A., Ghalab A.H. (2021). Removal of Syndesmotic Screw After Fixation in Ankle Fractures: A Systematic Review. Cureus.

[27] Steindel S.J. (2010). International classification of diseases, 10th edition, clinical modification and procedure coding system: Descriptive overview of the next generation HIPAA code sets. J. Am. Med. Inform. Assoc..

[28] Fonseca L.L.D., Nunes I.G., Nogueira R.R., Martins G.E.V., Mesencio A.C., Kobata S.I. (2017). Reproducibility of the Lauge-Hansen, Danis-Weber, and AO classifications for ankle fractures. Rev. Bras. De Ortop..

[29] Lins L., Carvalho F.M. (2016). SF-36 total score as a single measure of health-related quality of life: Scoping review. SAGE Open Med..

[30] Snaith R.P. (2003). The Hospital Anxiety And Depression Scale. Health Qual. Life Outcomes.

[31] Vahedi S. (2010). World Health Organization Quality-of-Life Scale (WHOQOL-BREF): Analyses of Their Item Response Theory Properties Based on the Graded Responses Model. Iran. J. Psychiatry.

[32] Huang C.T., Huang P.J., Lu C.C., Shih C.L., Cheng Y.M., Chen S.J. (2022). Syndesmosis Changes before and after Syndesmotic Screw Removal: A Retrospective Radiographic Study. Medicina.

[33] Pogliacomi F., Artoni C., Riccoboni S., Calderazzi F., Vaienti E., Ceccarelli F. (2018). The management of syndesmotic screw in ankle fractures. Acta Biomed..

[34] Kim J., Kwon M., Day J., Seilern und Aspang J., Shim J., Cho J. (2021). The Impact of Suture Button Removal in Syndesmosis Fixation. J. Clin. Med..

[35] Dao T.T., LeResche L. (2000). Gender differences in pain. J. Orofac. Pain.

[36] Dingemans S.A., Birnie M.F.N., Sanders F.R.K., Bekerom M.P.J.v.D., Backes M., van Beeck E., Bloemers F.W., van Dijkman B., Flikweert E., Haverkamp D. (2018). Routine versus on demand removal of the syndesmotic screw; a protocol for an international randomised controlled trial (RODEO-trial). BMC Musculoskelet. Disord..

[37] Briceno J., Wusu T., Kaiser P., Cronin P., Leblanc A., Miller C., Kwon J.Y. (2019). Effect of Syndesmotic Implant Removal on Dorsiflexion. Foot Ankle Int..

[38] Boyle M.J., Gao R., Frampton C.M.A., Coleman B. (2014). Removal of the syndesmotic screw after the surgical treatment of a fracture of the ankle in adult patients does not affect one-year outcomes. Bone Jt. J..

